# To promote or inhibit glioma progression, that is the question for IL-33

**DOI:** 10.15698/cst2021.01.240

**Published:** 2020-12-03

**Authors:** Stephen M. Robbins, Donna L. Senger

**Affiliations:** 1 Clark Smith Brain Tumour Centre, Arnie Charbonneau Cancer Institute, Department of Oncology, Cumming School of Medicine, University of Calgary, Calgary AB. T2N 4N1.

**Keywords:** brain tumor, interleukins, chemokines, tumor associated microglia, tumor associated macrophage

## Abstract

IL-33, a member of the IL-1 cytokine family has been shown to play a dual role within the body. First IL-33, similar to other IL-1 family members, is a secreted cytokine that binds to the cell surface receptor ST2 to induce a number of cell signaling pathways. Second, IL-33 enters the nucleus where it binds chromatin and directs transcriptional control of an array of growth factors and cytokines. Consistent with its complex cellular regulation, IL-33 mediates an array of biological functions by acting on a wide range of innate and adaptive immune cells. Recently, we found that IL-33 is expressed in a large number of human glioma patient specimens where its expression within the tumor correlates with the increased presence of Iba+ cells that include both resident microglia and recruited monocyte and macrophages. Strikingly, glioma derived expression of IL-33 correlates with a dramatic decrease in overall survival of tumor-bearing animals and thus supports its role as an influential factor in gliomagenesis. Notably however, when the nuclear localization function of IL-33 is crippled, the tumor microenvironment is programmed to be anti-tumorigenic and results in prolonged overall survival suggesting that when educated appropriately this could represent a novel therapeutic strategy for glioma (De Boeck *et al.* (2020), Nat Commun, doi: 10.1038/s41467-020-18569-4).

Glioblastoma represents one of the most fatal of human cancers. Although there has been progress in the treatment of glioblastoma as a result of a treatment regimen that combines maximal surgical resection with radiotherapy and concurrent and adjuvant temozolomide chemotherapy, two-year and five-year survival rates are only 25% and 10%, respectively, with average overall survival of less than 15 months from initial diagnosis. While there are several contributing factors including ineffective drug delivery across the blood-brain-barrier, complex cellular composition, diffuse invasiveness and the presence of chemo- and radio-resistant brain tumor-initiating cells [BTIC (a.k.a. glioma stem cells)], we propose that the unique microenvironment for which the glioma cells reside is a major contributor to the challenges of treating this disease. Specifically, the brain has a unique composition of cell types including neuronal and glial progenitors, neurons, astrocytes, oligodendrocytes, microglia/macrophages and brain endothelial cells all of which have been shown in some capacity to impact glioma growth and invasion. Often these non-neoplastic cell compartments can aid and abet by providing factors that promote survival, proliferation, and the invasive behaviour of the tumor cells.

Tumor-associated macrophages have been linked with high tumor grade and poor prognosis in many cancers including glioma. Microglia (brain resident macrophages) and infiltrated macrophages were first recorded in glioma tissue by Wilder Penfield in the mid 1920s. While the weighted importance of the resident microglia and the blood borne macrophages in glioma tumorigenesis is under debate, we propose that the ability to promote or inhibit glioma growth is context dependent and can be modulated when instructed appropriately. Using an extensive collection of patient-derived xenografts, we noticed that some glioma were highly inflammatory as denoted by the presence of macrophages and microglia. As a strategy to define factors that can determine this inflammatory tumor microenvironment we analyzed the tumor secretome from orthotopic xenografts with distinct inflammatory phenotypes. Using this approach, we identified a number of secreted factors which were either commonly or differentially expressed (**Figure 1**). Implementation of human or mouse specific antibodies provided the ability to attribute most of the identified proteins as either glioma-derived (human) or stromal-derived (murine) as highlighted in **Figure 1**. A number of secreted factors including IL-33, IP-10, IL-8 and SCF-1 were observed and may contribute to gliomagenesis in some manner. In our recent study we focussed on IL-33 based on its increasing relevance in tumorigenesis including glioma. We proposed that glioma-derived IL-33 orchestrates the brain tumor microenvironment by activating resident microglia and/or recruiting monocyte/macrophage innate immune cell populations from the cir-culation to promote gliomagenesis. Consistent with this, using both human and murine glioma models, we found that enforced expression of IL-33 results in the conversion of relatively immune inert glioma to a glioma with an immune rich environment that mediates rapid tumor growth and dramatic decrease in overall survival. In this context, IL-33 is both secreted by the glioma cells and present within its nucleus. Interestingly, when we ectopically expressed a mutant allele of IL-33 which lacked the nuclear localization signal (ΔNLS-IL33), this construct did not have an impact on glioma cell growth *in vitro,* but compromised the ability of the ΔNLS-IL33 expressing glioma cells to establish robust tumors when implanted intracranial. Assessment of the temporal progression of these tumors showed initial establishment of the tumor that was halted, or in some cases regressed, as evidenced by tumor remnants (i.e. glioma scar) detected by a glioma derived extracellular matrix protein known as tenascin-C. As indicated in the study, animals bearing tumors expressing the IL-33 crippled for nuclear localization showed significantly longer overall survival consistent with very low tumor burden. These observations thus raise one question: how does secreted IL-33 regulate a tumor environment that can result in either a pro-tumorigenic or an anti-tumorigenic phenotype?

**Figure 1:**
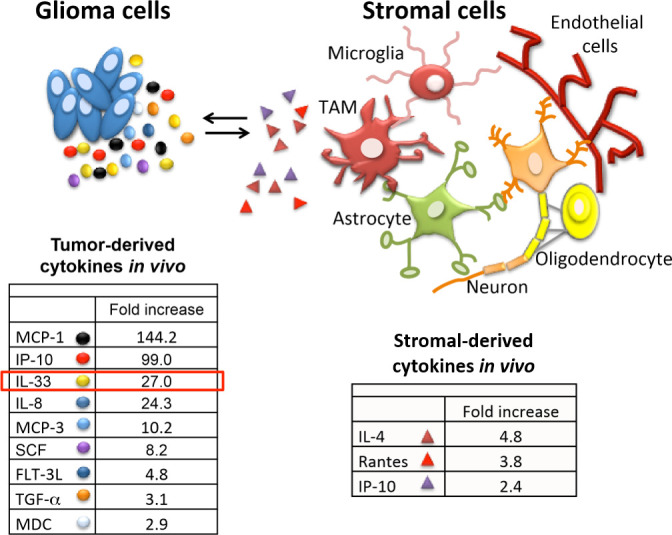
FIGURE 1: Schematic of the interplay between glioma cells and the host stromal environment. Tables show the presence of a number of cytokines including IL-33 increased in the interstitial fluid (secretome) of an inflammatory patient-derived glioblastoma xenograft when compared to an immune quiescent tumor. The tumor secretome was assessed using Luminex multiplex assays with human and mouse specific antibodies.

We propose the following model to explain this dramatic differential impact on glioma growth (**Figure 2**). In the context of wildtype IL-33 which is both in the nucleus of the glioma cell and secreted, we propose that nuclear IL-33 regulates or facilitates the expression of a large number of genes which includes several cytokines and chemokines. Together with secreted IL-33, these factors recruit innate immune cells from the circulation, and collectively activate both resident microglia and recruited monocytes/macrophages in a manner that acquires a protumorigenic phenotype (**Figure 2**). Consistent with this model, global gene expression analysis confirmed the expression of genes for a number of factors including IL8, CCL2, CXCL2 and CXCL3 with well-described chemokine functions. Furthermore, assessment of the immune repertoire within these glioma using single cell sequencing identified a number of distinct microglia and monocyte/macrophage cell states that include at least two populations of glioma-associated microglia and one distinct population of glioma-associated macrophages with features of a pro-tumorigenic M2-like phenotype. The exact weighted contribution between the microglia and recruited macrophages in promoting tumor progression awaits further mechanistic experimentation by targeting depletion of the various innate immune cell populations. One can, however, submit that both are involved at some level, and might be interchangeable if they can acquire similar phenotypes. In the context of secreted IL-33 alone, one can also speculate that IL-33 is still capable of activating resident brain cells such as microglia as well as innate immune cells from the periphery based on the presence of its cognate receptor. Moreover, in the absence of reprogramming of the tumor environment by nuclear IL-33, the innate immune cells may remain in a phenotype that is tumor suppressive and thereby maintain control of tumor growth.

**Figure 2:**
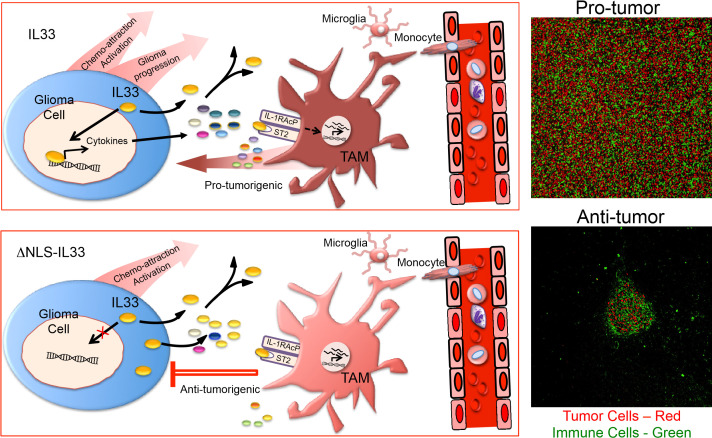
FIGURE 2: IL-33 nuclear function creates a switch-hitting cytokine in glioma progression. Schematics show the interplay between tumor associated macrophage (TAM) and glioma cells expressing full-length IL-33 (upper panel) or nuclear localization deficient IL-33 (ΔNLS-IL33; lower panel). Right panels show immunohistochemical images of brain sections from SCID mice bearing IL-33 (top) or ΔNLS-IL33 (bottom) expressing human glioma tumor cells co-stained for Iba-1 (green) to detect microglia/macrophage and anti-human nucleolin (red) to detect tumor cells.

Thus, if one could recapitulate the tumor microenvironment when the nuclear localization of IL-33 is crippled, it may provide an alternative or additional therapeutic strategy for treating glioma patients. In other words, changing the treatment strategy from solely targeting glioma cells to a therapeutic strategy that includes modulation and reprogramming of the patients own immune system to help control tumor growth might provide therapeutic benefits.

However, there are still a number of unanswered questions, i.e. how is IL-33 secreted from the glioma cells? Secretion of IL-33 from glioma cells is minimal *in vitro* and was only observed when the tumor cells were implanted *in vivo*. Originally, IL-33 was identified as an alarmin, and as the name suggests, is released from damaged or injured cells. While it is possible that IL-33 is released from dying cancer cells in the necrotic regions of tumors, we do not see major signs of necrosis in our models especially when tumor burden is very small. Instead, our data suggests that IL-33 is released via regulated secretion and we speculate that IL-33 in addition to being secreted might also be released by exosomes, a route that raises the possibility of IL-33 signaling independent from and in addition to the ST2 receptor. Moreover, emerging evidence supporting a role for IL-33 in a number of cancers together with its potential to reprogram the tumor immune environment raises the obvious question on the impact of IL-33 in current and future cancer therapies. One may speculate that some treatments such as radiation/chemotherapy may enhance the release of IL-33 and thus lead to unintended consequences that further promote a pro-tumorigenic environment. Likewise, orchestration of the cellular environment could have a profound effect on the potential success of immune-based or oncolytic-based therapies. Whether this effect provides an advantage or a barrier for the treatment needs further investigation. These are interesting times for an interesting cytokine.

